# Structural architecture of the human long non-coding RNA-PAAN as a potential target for anti-influenza drug development

**DOI:** 10.1016/j.jbc.2025.110755

**Published:** 2025-09-23

**Authors:** Izabela Ulanowska, Ryszard Kierzek, Elzbieta Kierzek, Marta Soszynska-Jozwiak

**Affiliations:** Institute of Bioorganic Chemistry, Polish Academy of Sciences, Poznan, Poland

**Keywords:** long noncoding RNA, RNA secondary structure, influenza virus, RNA virus, negative–strand RNA virus, host–pathogen interaction, antisense oligonucleotides

## Abstract

Influenza A virus (IAV) is considered one of the most dangerous pathogens in the world because of its great variability. Available anti-influenza drugs suffer from the rapid emergence of drug resistance. Thus, there is an urgent need to develop new antiviral strategies with new mechanisms of action and reduced drug resistance potential. Host lncRNAs are much less variable than influenza RNA/protein; therefore, they can be a new and universal aim of antiviral therapy. Recently, studies have revealed the interferon-independent host lncRNAs-PAAN that interacts with the influenza virus PA protein to promote viral replication. Until now, data concerning the lncRNA-PAAN structure *in vitro* and in biological environments have not been discovered. Here, for the first time, we used chemical mapping and the SHAPE method to propose the secondary structure of lncRNA-PAAN *in vitro* and in the cellular environment. We discuss the experimental and computational approaches that have led to distinct structural models. Finally, we defined the structural motifs of lncRNA-PAAN of potential functionality forming during influenza A infection and designed lncRNA-PAAN structure-specific antisense oligonucleotides (ASOs). Several of these ASOs significantly lowered the level of lncRNA-PAAN and inhibited IAV infection. Our findings not only advance our understanding of the complexity of the IAV-host interactions but also could be used for designing a new anti-influenza A strategy targeting the host lncRNAs.

Influenza A virus (IAV) is considered one of the most dangerous pathogens in the world (1, 2, 3, 4). Because of its ability to reassort, it results in adaptation to new host environments, evasion of host immune responses, and acquisition of resistance to antiviral drugs (5, 6). Moreover, the influenza virus shows high mutation rates caused by viral polymerase, resulting in dramatic changes in its pathogenicity (5, 6). Vaccination is the most popular strategy against influenza (7); however, its efficacy levels can vary between seasons (8). Anti-influenza drugs suffer from losing their effectiveness (9), so there is a great need to find other methods for controlling the influenza virus.

Host long non-coding RNAs (lncRNAs) necessary for the influenza replication cycle are much less variable than influenza RNAs or proteins (9); therefore, they can be a new and universal target of antiviral therapy. LncRNAs are characterized by more than 200 nucleotides in length, which do not encode proteins but are subject to RNA maturation processes characteristic of coding transcripts. In addition, they interact with DNA, RNA, and proteins and have essential biological functions such as regulation of transcription, protein synthesis, intron excision, and cytoplasmic or nuclear transport (10, 11, 12, 13, 14, 15). A change in the expression profile of lncRNAs in mammalian cells during various diseases, including those caused by the influenza virus, has been observed (16). Recent studies have identified lncRNAs acting as regulators of host-virus interactions during viral infection (17, 18, 19, 20, 21, 22, 23). Most lncRNAs regulate virus–host interactions by activating interferon (IFN) and cytokine production, interferon-stimulated genes (ISGs), and pattern recognition receptor-associated (PRR) signaling. Some are also associated with the activation of transcription factor expression, such as the nuclear factor κ-light-chain-enhancer of activated B cells (NF-κB) (21, 24, 25, 26, 27). For example, IAV-induced lncRNA NRAV is a negative regulator of innate antiviral immunity by repressing interferon-stimulated gene transcription (28). The lncRNA TSPOAP1-AS1 negatively modulates type I interferon signaling to promote IAV replication (29). Lnc-MxA inhibits INF-β transcriptional activation by forming an RNA–DNA triplex on its promoter (30). Lnc-Lsm3b prevents the overproduction of type I interferon and promotes IAV replication (31). Some host lncRNAs are not induced by interferon during IAV infection. For example, the lncRNA ACOD1 induced by IAV and many other viruses increases the metabolic activity of the enzyme GOT2 (aspartate aminotransferase), which alters host metabolism, favorably affecting virus replication (32). More recent studies have revealed the interferon-independent host lncRNAs IPAN, PAAN (PA-associated noncoding RNA), and VIN (22, 23, 33). It has been shown that lncRNA-IPAN binds to the viral protein PB1 and prevents PB1 degradation, thereby promoting IAV transcription and replication (23). IAV also upregulates lncRNA-VIN; loss of lncRNA-VIN results in reduced IAV production and inhibits viral protein synthesis, suggesting that lncRNA-VIN is essential for productive IAV infection (33). The IAV specifically captures lncRNA-PAAN to promote its replication. It was found that lncRNA-PAAN binds to the viral PA protein and promotes viral RNA polymerase complex formation, which affects efficient vRNA synthesis. The human lncRNA-PAAN gene (LOC100506319) is on chromosome 3p21.31 and encoded by four exons (22). To date, structural studies of the host lncRNA-PAAN are still largely undiscovered.

In this study, we propose for the first time the secondary structure of lncRNA-PAAN *in vitro* and in the cellular environment. Finally, we defined the structural motifs of potential functionality and designed lncRNA-PAAN structure-specific antisense oligonucleotides (ASOs). Several of these ASOs significantly lowered the level of lncRNA-PAAN and inhibited IAV infection. Our findings not only advance our understanding of the complexity of the IAV–host interactions but also could be used for designing a new anti-influenza A strategy targeting the host lncRNA.

## Result and discussion

### Computational predictions of potential functional RNA structures of lncRNA-PAAN

The current model is that lncRNA's function depends on a specific, typically short, conserved motif, divided by regions where specific sequences and/or structures are less critical (34). Moreover, there is some evidence from experimentally solved structures of conserved secondary structure motifs, for example, in MALAT1 (35, 36), XIST (37, 38), Cyrano (39), MEG3 (40, 41), and COOLAIR (42).

Importantly, functional noncoding (nc) RNAs structures have lower (more stable) minimum free energy (MFE) values than random sequences with the same nucleotide content (43, 44). Therefore, we used ScanFold 2.0, which focuses on a single RNA sequence and relies on the z-score (calculated by comparing the predicted MFE of a sequence with the average MFE of matched randomized sequences with the same nucleotide content), to identify potentially functional motifs within lncRNA-PAAN (45). Negative z-scores indicate enhanced thermodynamic stability relative to random RNAs with the same length/nucleotide content, a key feature of functional RNA structures. Notably, ScanFold was able to successfully identify and model the structures of known functional motifs, such as the Rev Response Element of the HIV-1 virus, which binds to the Rev protein (46).

**Figure 1:**
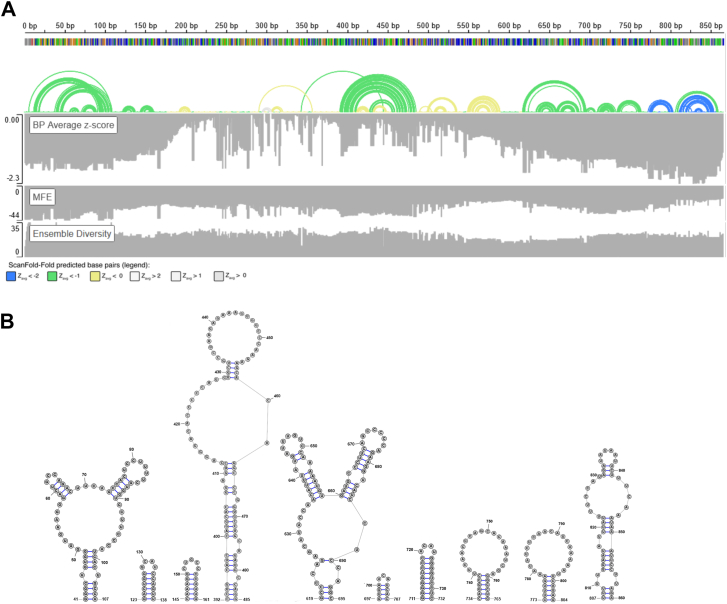
**ScanFold analysis of the lncRNA-PAAN.***A*, an IGV visualization of ScanFold data for the lncRNA-PAAN. A positional marker is marked at the top every 50 base pairs. Below this is the base pair track resulting from ScanFold-Fold. Next is the z-score track, the MFE track, and the ensemble diversity track from the ScanFold-Scan analysis. *B*, ScanFold RNA 2D structures are shown for the lncRNA-PAAN. Structures have been visualized here using the RNA secondary structure Editor.

For lncRNA-PAAN, the predicted MFE across all windows ranged from −15.40 to −43.74 kcal/mol and averaged −29.33 kcal/mol (Fig. 1*A*). The z-score across the lncRNA-PAAN sequence ranged from −2.29 to 0, with a local region having a highly negative z-score (<−2) (Fig. 1*A*). ScanFold models regions likely to form structure and define unique motifs. For each bp average thermodynamic z-score for the most favorable arrangement is calculated (Zavg). ScanFold identified ten local motifs with a high likelihood of functionality predicted in the region of lncRNA-PAAN: 41 to 107 nts, 123 to 138 nts, 145 to 161 nts, 392 to 485 nts, 619 to 695 nts, 697 to 707 nts, 711 to 732 nts, 734 to 765 nts, 773 to 804 nts, 807 to 860 nts (Fig. 1*B*). Motifs 773 to 804 and 807 to 860 nts were identified with at least Zavg < −2, and the remaining structures are characterized by at least Zavg < −1 (Fig. 1*A*). These motifs are localized mainly at lncRNA-PAAN's 5′ and 3′ ends; however, a lower Z-score characterizes the 3′-end of lncRNA-PAAN structures.

### Chemical mapping of lncRNA-PAAN *in vitro*

Most RNA motifs in lncRNAs have been studied using chemical and enzymatic probing (34). These methods were applied to MEG3 (40), XIST (47, 48), CYRANO (39), SRA (49), HOTAIR (50), COOLAIR (42), Braveheart (51), MALAT1 (35, 52), PAN (53), ROX1, and ROX2 lncRNA (54). Therefore, we used chemical mapping to expand our knowledge about the secondary structure of lncRNA-PAAN and verify our prediction performed by the ScanFold 2.0 program. To assess flexible, single-stranded regions of lncRNA-PAAN, we used three reagents: DMS (dimethyl sulfate, methylates N1 of A and N3 of C), CMCT (1-cyclohexyl-(2-morpholinoethyl) carbodiimide metho-p-toluene sulfonate, acylates N1 of G and N3 of U), and NMIA (N-methylisatoic anhydride, acylates 2′-OH of accessible nucleotides). Before each experiment, lncRNA-PAAN was folded in a folding buffer (300 mM NaCl, 5 mM MgCl_2_, 50 mM HEPES, pH 7.5) to obtain properly folded RNA (Fig. S1). As mapping results, we observed that DMS strongly methylated 117 nts (24% of A/Cs), CMCT acylated 110 nts (29% of U/Gs), and NMIA strongly modified 138 nts and moderately modified 74 nts, representing 24% of nucleotides (Table S1). These results were complementary and revealed six regions in lncRNA-PAAN with high flexibility (≥7 flexible nts): 78 to 84 nts, 374 to 386 nts, 437 to 444 nts, 779 to 785 nts, and 791 to 805 nts.

### Modular folding and conformational dynamics of lncRNA-PAAN *in vitro* revealed through integrative chemical mapping

**Figure 2:**
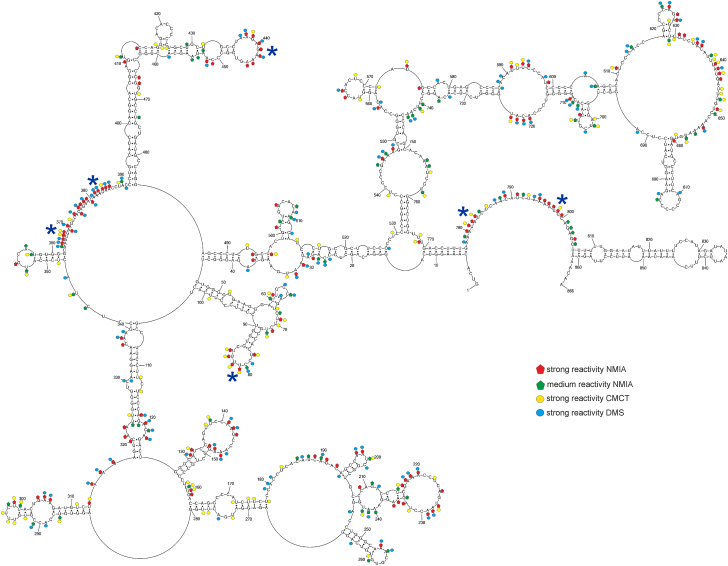
***In vitro*****model of MFE secondary structure of lncRNA-PAAN.** This global folding model was predicted by RNAstructure 6.4, based on *in vitro* chemical probing with DMS, CMCT, and NMIA reactivity. Strong (≥0.7) and medium (0.7–0.5) reactivities of NMIA, as well as strong hits of DMS and CMCT, are marked on the structure. *Blue asterisks* mean flexible regions with at least seven modified nucleotides.

**Figure 3:**
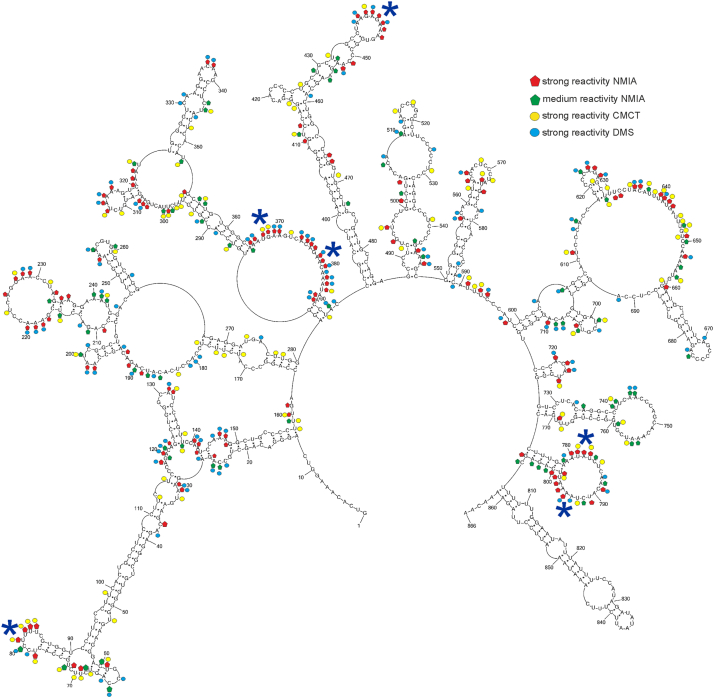
**Local secondary structure model of lncRNA-PAAN mapped *in vitro.*** Local folding of lncRNA-PAAN predicted by RNAstructure 6.4 using a maximum pairing distance mode and experimental data from *in vitro* probing experiments. The structure was predicted by applying strong DMS and CMCT modifications, SHAPE reactivities converted to pseudo-free energies, and a maximum pairing distance of 150 nucleotides. Strong (≥0.7) and medium (0.7–0.5) reactivities of NMIA and strong hits of DMS and CMCT are marked on the structure. *Blue asterisks* mean flexible regions with at least seven modified nucleotides.

To predict the secondary structure of lncRNA-PAAN, SHAPE data were used as pseudo-energy constraints, and strong DMS and CMCT modifications were employed as chemical mapping constraints in the RNAstructure 6.4 program. Global and local MFE structures were modeled to reflect both long-range and short-range interactions, which are characteristic of lncRNAs (36, 55). We also employed the Maximum Expected Accuracy (MEA) predictions to assess how the folding algorithm and approach influence the prediction (56, 57). Our global MFE model (ΔG°_37_ = −414.0 kcal/mol) consists of helices with plenty of accessible bulges and loops (Fig. 2). We showed that most accessible regions defined by chemical mapping correspond to single stranded regions. The 5′ and 3′ ends of the lncRNA-PAAN model are single-stranded. Several long-distance interactions were identified: 5 to 12/770 to 777 nts, 14 to 29/515 to 526 nts, 35 to 45/486 to 96 nts, 104 to 126/317 to 341 nts, and 531 to 537/760 to 766 nts. The local MFE model (ΔG°_37_ = −387.1 kcal/mol) predicted 11 local motifs and preserved four hairpins from the global structure (163 to 280 nts, 392 to 485 nts, 599 to 715 nts, 806 to 861 nts; Fig. 3).

**Figure 4:**
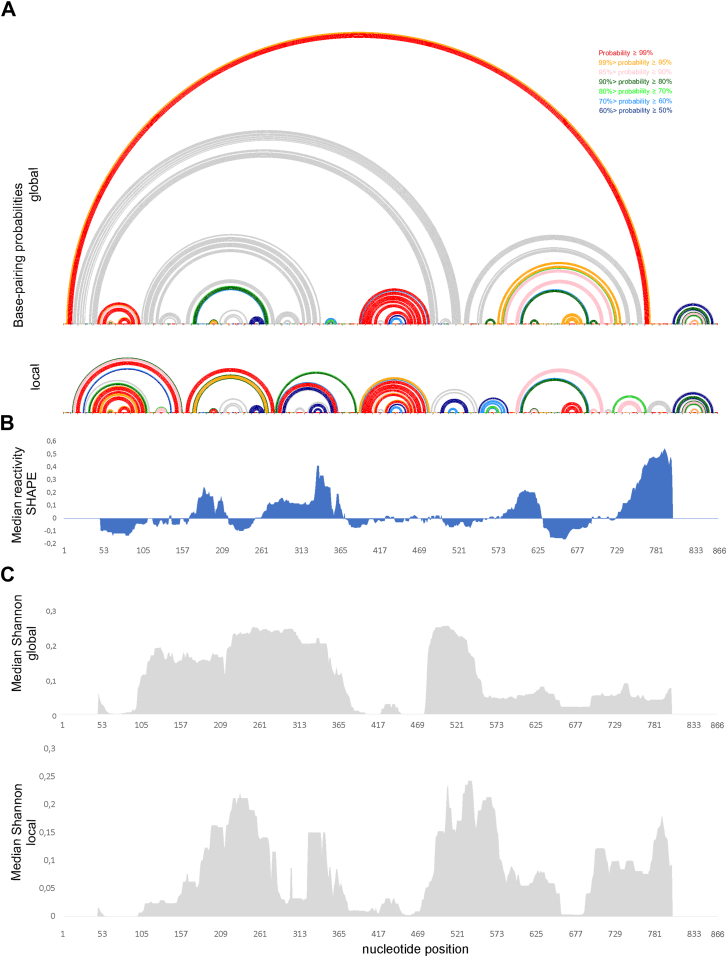
**Global and local secondary structure features of lncRNA-PAAN determined*****in vitro.**A*, Arc plots showing the base-pairing probabilities of predicted MFE global (*upper*) and local (*lower*) structures probed *in vitro.* The colors indicate the percentage of pairing probability. The probability lower than 50% is colored *grey*. *B*, Median SHAPE reactivities were calculated in a 50 nt window and plotted with respect to the global median. *C*, median Shannon entropies of MFE global (*upper*) and local (*lower*) structures were calculated in a centered, sliding 50-nucleotide window. Regions of 50 nts from 5′ end and 60 nts from 3′ end of lncRNA-PAAN (graphs: median Shannon, and median reactivity) were excluded from visualization.

It is known that highly probable pairs are more likely to be correctly predicted pairs (58). Therefore, base-pairing probabilities were computed using partition function analysis, incorporating experimental constraints (59). Results show that there are several regions exhibited high-probability (≥90%) base pairs or unpaired nucleotides in both global and local models, *e.g.*, 46 to 102, 194 to 205, 392 to 394/483 to 485, 396 to 415/460 to 480, 426 to 429/456 to 459, 599 to 603/711 to 715, 660 to 687, 820 to 822/847 to 849, and 825 to 844 nts (Fig. 4), suggesting the consistent formation of these motifs *in vitro.*

To further evaluate structural confidence, MEA models were generated (Figs. S2 and S3). Generating a structure composed of highly probable base pairs is an alternative method for structure prediction that may have higher fidelity (59). The MEA local structure (Fig. S3) was highly similar to the MFE structure (Fig. 3). The most significant differences in global MEA and MFE concern the regions: 14 to 45/486 to 526 nts, 104 to 126/317 to 341 nts, 127 to 162 nts, 163 to 177/267 to 280 nts, 207 to 244 nts, 531 to 554/745 to 766 nts; while other motifs remained identical.

To continue exploring local and global folding of lncRNA-PAAN, we used a shotgun approach to identify potential independently-folded sub-domains (60). We divide the lncRNA-PAAN into five overlapping fragments and probe these fragments (Table S2). Fragments F1 and F2 span the entire lncRNA-PAAN sequence without overlapping. Fragment F3–F5 overlaps with those of F1 and F2. We subsequently analyze the probing profiles of the fragments alongside those of the complete RNA to identify potential self-folding sub-domains. Sequence regions with comparable profiles in both the fragment and full-length RNA contain only base pairing partners within the fragment. The shotgun probing approach reveals modular subdomains within nucleotides 14 to 156, 284 to 364, 392 to 485, 660 to 687, and 806 to 861. Notably, both MFE and MAE structural models independently predict motifs within these regions. These results provide strong support for stable local base pairing in the predominance of local base pairing over long-range interactions in structural models of lncRNA-PAAN. This pattern can be functionally relevant, potentially enabling the RNA to adapt specific domains for interactions with distinct protein or nucleic acid partners. The identification of independently folding subdomains within lncRNA-PAAN—confirmed both by shotgun probing and base-pairing probability analyses—mirrors similar organizational principles reported for paradigmatic lncRNAs such as MALAT1, XIST, and HOTAIR (61). For instance, studies on XIST have demonstrated modular domains responsible for distinct functions, such as gene silencing and RNA localization, often stabilized by local intramolecular interactions rather than global compaction (38). Likewise, the HOTAIR lncRNA contains structured modules that mediate interactions with both Polycomb Repressive Complex 2 (PRC2) and LSD1/CoREST/REST, with limited long-range pairing (50).

Subsequently, we use Rsample, which integrates experimental data with thermodynamic modeling to estimate structurally diverse RNA ensembles *via* Boltzmann sampling (62). Rsample predicted four alternative secondary structures of lncRNA-PAAN (Fig. S4), showing long distance and local interactions are dynamic and influence secondary structure formation. Interestingly, that region 170 to 641 nts. is the most changeable (Fig. S4). However, changes in secondary structure are mainly local.

To validate our results, we used integrative probing analysis of nucleic acids empowered by multiple accessibility profiles (IPANEMAP) (63). Unlike RNAstructure, which predicts based on the minimization of free energy, IPANEMAP models the RNA structural ensemble and identifies representative conformations that reflect RNA structural flexibility. The IPANEMAP model (Fig. S5) exhibits a high density of long-range interactions, with local structural motifs spanning nucleotide positions 194 to 205, 660 to 687, and 806 to 861, which are also present in the MFE and MAE models. Together, these data support a model in which lncRNA-PAAN adopts a semi-modular architecture composed of multiple independently folding subdomains embedded within a flexible scaffold. This organization could facilitate both structural stability and dynamic adaptability, features commonly associated with the regulatory functions of long non-coding RNAs (64).

### Chemical mapping of lncRNA-PAAN in the cellular environment

The previous study showed that lncRNA-PAAN expression was profoundly increased by IAV infection in HEK293T, A549, and SupT1 cells (22). To investigate its secondary structure under cellular conditions, we used lysates from infected A549 cells and confirmed viral protein expression *via* Western blot. (Fig. S6*B*). The amount of viral RNA copies was analyzed by RT-qPCR (Fig. S7). These lysates preserve endogenous components—proteins, RNA, DNA, and lipids—thus mimicking an *in vivo*-like environment (65). This system enables chemical probing of low-abundance RNAs (65) such as lncRNA-PAAN and permits the use of membrane-impermeable reagents like CMCT. Accordingly, we applied the same mapping reagents as *in vitro*. For our lysate mapping experiments, we utilized human 18S ribosomal RNA (rRNA) as a control. The 18S rRNA is part of the small ribosomal subunit and possesses a well-characterized structure. We compared the modification sites identified in our experiments (Table S3) with the determined secondary structure of 18S rRNA in the human ribosome (RNAcentral database, ID: URS00005A14E2_9606 (https://rnacentral.org/rna/URS00005A14E2/9606)). The modification sites for 18S rRNA were located within regions that are single-stranded and accessible to solvent, confirming the reliability of the lysate probing system.

Our mapping data of lncRNA-PAAN showed that DMS strongly modified 109 nucleotides (22% of A/Cs), CMCT probed 123 nucleotides (32% of U/Gs), and NMIA strongly or moderately modified 199 nucleotides (23% of all nucleotides) (Table S3). The resulting modification patterns were consistent across reagents and revealed seven highly accessible regions (each ≥7 modified nucleotides): 78 to 84 nts, 215 to 221 nts, 223 to 229 nts, 372 to 378 nts, 380 to 386 nts, 623 to 632 nts, and 793 to 800 nts.

### Mapping in the cellular environment confirms the modular folding and structural dynamics of lncRNA-PAAN

**Figure 5:**
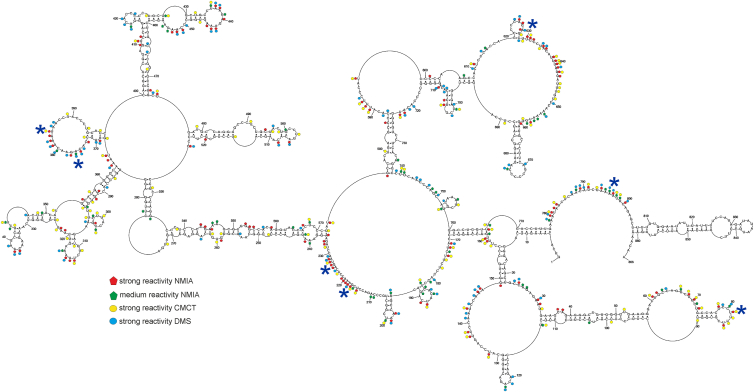
**Model of the MFE secondary structure of lncRNA-PAAN mapped inthe cellular environments.** This model was predicted by RNAstructure 6.4 using experimental mapping data from the cellular environments. Strong (≥0.7) and medium (0.7–0.5) reactivities of NMIA and strong hits of DMS and CMCT are marked on the structure. *Blue asterisks* mean flexible regions with at least seven modified nucleotides.

**Figure 6:**
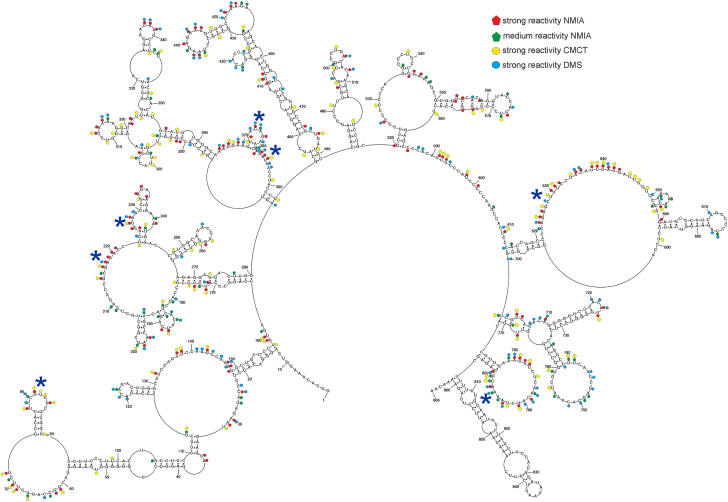
**Local secondary structure of lncRNA-PAAN determined in the cellular environments.** A local model of lncRNA-PAAN as predicted by RNAstructure 6.4 using a maximum pairing distance of 150 nucleotides and experimental chemical mapping data from the cellular environments. Strong (≥0.7) and medium (0.7–0.5) reactivities of NMIA, and strong hits of DMS and CMCT are marked on the structure. *Blue asterisks* mean flexible regions with at least seven modified nucleotides.

**Figure 7:**
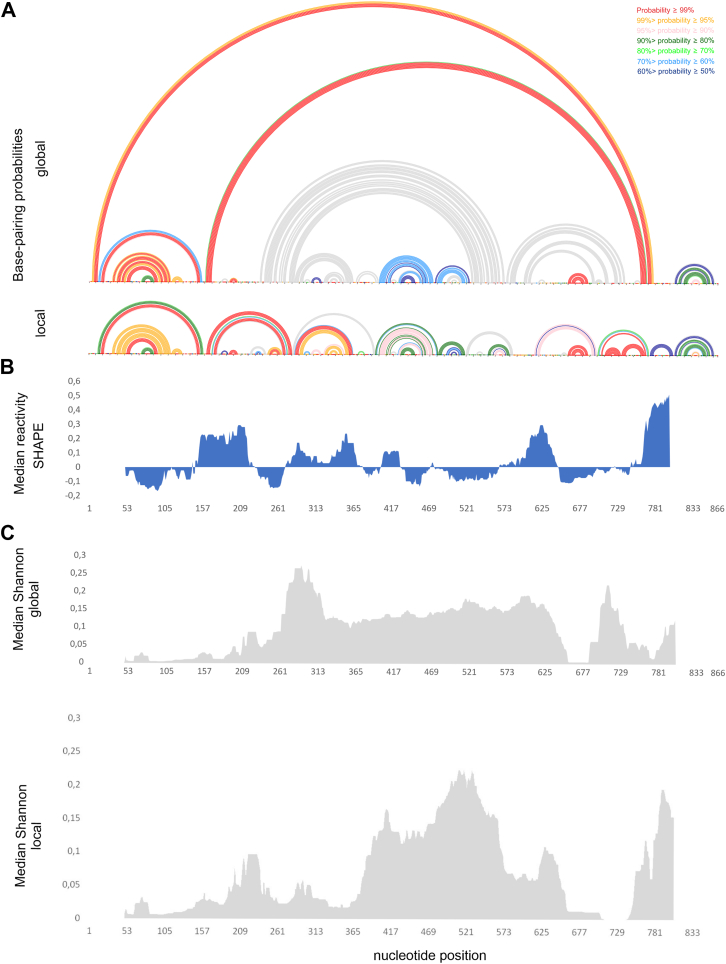
**Global and local secondary structure features of lncRNA-PAAN*****in cellular environments.**A*, ARC plots showing the base-pairing probabilities of predicted MFE global (upper) and local (lower) structures probed in cellular environments. The colors indicate the percentage of pairing probability. The probability lower than 50% is colored *grey*. *B*, median SHAPE reactivities were calculated in a 50 nt window and plotted with respect to the global median. *C*, median Shannon entropies of MFE global (*upper*) and local (*lower*) structures were calculated in a centered, sliding 50-nucleotide window. Regions of 50 nt from the 5′ end and 60 nt from the 3′ end of lncRNA_PAAN (graphs: median Shannon, and median reactivity) were excluded from visualization.

To predict the secondary structure of lncRNA-PAAN in infected A549 cell lysate, we introduced experimental data to the RNAstructure 6.4 program in the same manner as *in vitro* prediction. Moreover, we also considered local and global MFE structures. The global MFE model (ΔG°_37_ = −400.5 kcal/mol) exhibits multiple canonical RNA elements, including hairpins, internal loops, bulges, and helices (Fig. 5). Notably, the 5′ and 3′ ends remained unpaired, and several long-range interactions were predicted between nucleotide regions: 5 to 12/770 to 777 nts, 161 to 169/760 to 768 nts, 236 to 283/527 to 572 nts. On the contrary, the local structure (ΔG°_37_ = −371.6 kcal/mol) created 11 hairpins of which three were also formed using the global folding option: 13 to 158 nts, 482 to 518 nts, and 806 to 861 nts. A fourth hairpin, spanning 400 to 474 nts in the global model, was extended in the local prediction to 395 to 481 nts (Fig. 6). Both models are consistent with the probing results: single-stranded regions are extensively modified, while base-paired helices exhibit minimal reactivity. Using chemical constraints, we calculated base-pairing probabilities for both structures. Results demonstrated several regions of both models with paired and unpaired nucleotides of more than 90% probability: 33 to 57/91 to 113 nts, 115 to 129 nts, 194 to 205 nts, 660 to 687 nts, and 826 to 844 nts (Fig. 7), indicating these structural motifs are likely present in the cellular context. Additionally, global and local MEA structures of lncRNA-PAAN were predicted using experimental constraints (Figs. S8 and S9). The MEA local structure (Fig. S9) was highly similar to the MFE counterpart (Fig. 6). The global MFE and MEA are dissimilar, and the main differences appear in the regions: 236 to 281/527 to 572 nts, 284 to 295/354 to 364 nts, 324 to 353 nts, 365 to 399 nts, 575 to 587/724 to 738 nts, 599 to 608/693 to 715 nts (Figs. 5 and S8). The MFE and MEA models support existence motifs in the region of lncRNA-PAAN: 46 to 56/91 to 102 nts, 68 to 90 nts, 194 to 205 nts, 400 to 415/460 to 474 nts, 426 to 429/456 to 459 nts, 660 to 687 nts, 806 to 861 nts. The shotgun approach also supported these regions. To better understand the structural dynamics of lncRNA-PAAN, we performed RNA secondary structure ensemble analysis using Rsample (62). The mixture of infected, bystander, and unaffected cells may also contribute to the heterogeneity of the RNA in the cell lysate. The resulting ensemble (Fig. S10) revealed dynamic regions capable of adopting multiple conformations. These data are consistent with the results obtained for *in vitro* mapping, indicating a modular architecture characterized by multiple well-defined local motifs embedded within a structurally flexible scaffold. Importantly, the IPANEMAP model (Fig. S11) independently identified key structural motifs in regions 194 to 205, 660 to 687, and 806 to 861 nts. These motifs were also supported by the MFE, MEA, and Rsample models and closely mirrored the in vitro-derived secondary structure, strengthening the biological relevance of the predicted RNA fold. Together, these results suggest that lncRNA-PAAN adopts a modular secondary structure composed of well-defined motifs interspersed with conformationally dynamic regions, which may facilitate structural rearrangements or functional switching.

### Identified structural motifs of lncRNA-PAAN with high fidelity

**Figure 8:**
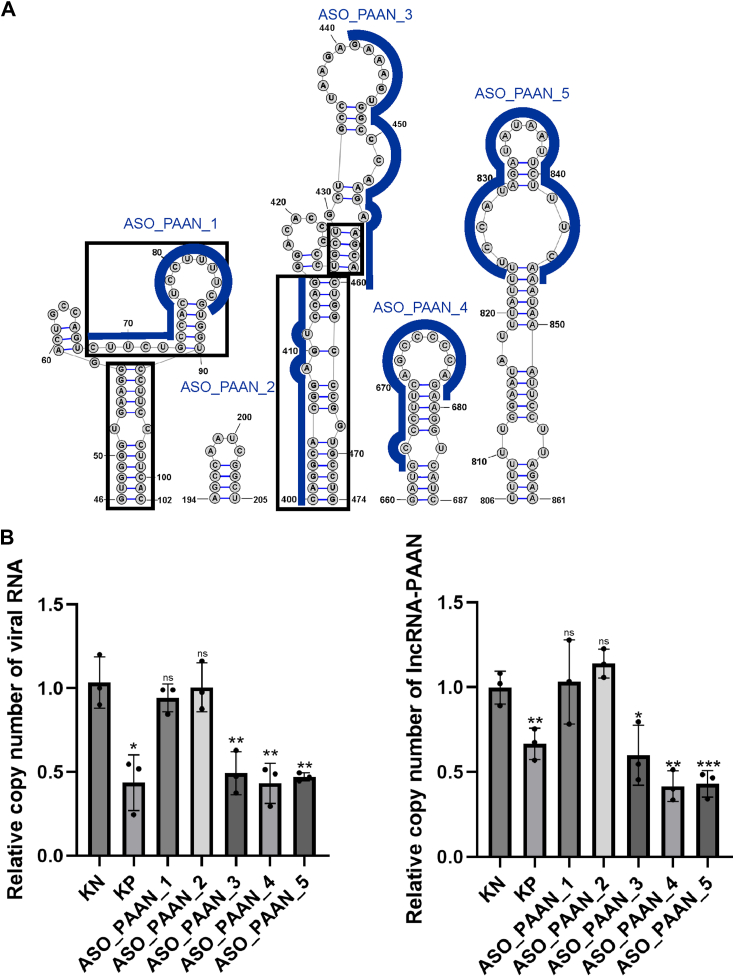
**High-fidelity prediction of lncRNA-PAAN structural motifs and their functional analysis in influenza A virus–infected cells**. *A*, structural motifs predicted in lncRNA-PAAN with high fidelity using experimental data. The *black rectangle* on motifs 46 to 102 nts. and 400 to 474 nts. indicate structures determined with high fidelity; the remaining regions of these two motifs are uncertain. Target regions for designed ASOs are marked with *blue lines*. *B*, quantitative analysis of viral RNA and lncRNA-PAAN by real-time PCR in A549 cells infected by influenza A virus. RNA copy number of ASO-treated samples was compared to the RNA copy number of the negative control KN (established as 1). Bars represent the mean ± standard deviation (SD) of n = 3 biological replicates. Each dot represents an independent biological replicate. The unpaired two-tailed Student's *t* test was performed for statistical comparisons (∗*p* < 0.05, ∗∗*p* < 0.01, ∗∗∗*p* < 0.005, ns - not significant).

LncRNA-PAAN plays an essential role during the replication cycle of the influenza A virus. It is established that the functions of lncRNA depend on the ability to fold into thermodynamically stable secondary and higher-order structures (66). Our previous analyses identified distinct thermodynamically stable structural motifs in regions 46 to 102, 400 to 474, 194 to 205, 660 to 687, 806 to 861 nts within lncRNA-PAAN that are preserved across both *in vitro* and in cellular environments (Fig. 8). Interestingly, some functional RNA motifs fold into the same secondary structure both *in vitro* and *in vivo*, for instance, hairpins SL1, SL2, and SL3 of the 5′UTR of SARS-CoV-2 (67, 68, 69, 70). Furthermore, motifs spanning regions 46 to 102 and 660 to 687 nts were characterized by overlapping profiles of low SHAPE reactivity and low Shannon entropy parameters (Figs. 4 and 7) that together indicate the presence of well-determined, low-ensemble-diversity RNA folds (59). Motif 400 to 474 nts is characterized by low SHAPE reactivity and Shannon entropy *in vitro,* while in the cellular environment, these parameters are higher. The increase in Shannon entropy of the motif in the cellular environment, relative to its low-entropy, low-SHAPE conformation *in vitro*, suggests a transition toward a more structurally dynamic state. This shift may reflect RNA-protein interactions or regulatory structural plasticity, consistent with previous studies demonstrating environment-dependent modulation of RNA folding (71, 72). Despite the lack of experimental mapping data for the 806 to 861 nts region, its recurrent presence under all tested conditions indicates that this structural motif is stable thermodynamically. In addition, newly identified motifs of lncRNA-PAAN are characterized by a high base pair probability in all presented models (only base pair probability for motif 400–474 nts mapped in lysate is at the level of 60–70%). The preservation of these motifs suggests that they may be formed in cells during IAV infection and are essential for viral replication.

### lncRNA-PAAN secondary structure could be useful to design antivirals

To explore the role of well-defined, predicted RNA motifs of host lncRNA-PAAN in IAV replication, we designed four ASOs (Table S4). Oligonucleotides were designed to target mainly single-stranded regions and were fully complementary to lncRNA-PAAN (Fig. 8*A*). In the creation of ASOs, 2ʹO-methylated RNA and locked nucleic acid (LNA) nucleotides were utilized to enhance their thermodynamic and cellular stability (73). The targets spanned regions 68 to 86, 441 to 460, 663 to 679, and 824 to 846 nts. Additionally, we used a negative control (scrambled ASO, not complementary to lncRNA-PAAN and IAV sequences) and ASO_PAAN_2, which targets mainly double stranded motif (400–415 nts). As a positive control, we used an ASO targeting the conserved secondary structure motif of segment two of IAV because no single ASO targeting lncRNA PAAN is known. We did not design the ASO to target the hairpin 194 to 205 because single stranded region is too short to bind to ASO effectively.

All ASOs were tested in A549 cells infected with the influenza A virus. Three of the four tested showed significant inhibition of IAV replication, as determined using quantitative real-time PCR (RT-qPCR) compared to cells transfected with a negative control (Fig. 8*B*). ASO_PAAN_4 and 5 inhibited IAV replication by 57% and 53%, respectively. ASO_PAAN 3 decreased viral RNA level by 51%. The ASO_PAAN_1 showed no significant inhibitory effect on IAV replication. It is possible that the region that is targeted by ASO_PAAN_1 forms noncanonical RNA motifs or takes part in tertiary interactions, causing inaccessibility to ASO. As expected, ASO_PAAN_2 did not inhibit IAV replication. All tested ASOs were checked for cytotoxicity in A549 cells using CellTiter-Glo Assay. No effect on cell viability was observed at 750 nM, the concentration used for evaluating antiviral activity (Fig. S12). We then examined the impact of ASOs on the level of lncRNA-PAAN. Through RT-qPCR analysis, we found that all ASOs capable of inhibiting IAV infection reduced the level of lncRNA-PAAN in A549 cells (Fig. 8*B*). The level of silencing lncRNA-PAAN by ASO PAAN_4 and _5 is similar to the effect obtained by secondary structure independent lncRNA-PAAN knockdown using esiPAAN in A549 cells (22), showing their effectiveness. As expected, the ASO designed to target the double-stranded lncRNA-PAAN motif did not affect the lncRNA-PAAN level. Interestingly, we also observed a decrease in lncRNA-PAAN level in the case of positive control targeting viral RNA. This is possible because IAV induces lncRNA-PAAN, and the infection level depends on it. In other words, reducing the IAV replication by ASO resulted in lower lncRNA-PAAN levels.

We found a relationship between the interference of lncRNA-PAAN expression and the antiviral efficacy of ASOs, confirming that the silencing of lncRNA-PAAN by ASOs is a key factor in their antiviral activity. Together, these data demonstrate that motifs of 400 to 474 nts, 660 to 687 nts, and 806 to 861 nts. of lncRNA-PAAN are required for efficient IAV replication.

Our ASOs were designed to steric-block or influence lncRNA-PAAN folding. Hence, we checked if our ASOs change the secondary structure of lncRNA-PAAN (we selected ASO_PAAN_3 and ASO-PAAN_4 for those experiments; ASO_PAAN_5 was omitted because its target region was outside the mapping reading region). The mapping results of lncRNA-PAAN with ASO were consistent with the secondary structure of lncRNA-PAAN (strong reactivities ≥ 0.7, medium 0.7–0.5, and weak ≤0.5 remained in the same range) (Table S5). We observed only reverse transcription stops in the target region of ASOs, indicating an interaction between the ASO and its target region. Therefore, ASO_PAAN_3, 4, and 5 have their effect through steric blockade of lncRNA-PAAN. Motifs 400 to 474 nts, 660 to 687 nts, and 806 to 861 may be responsible for interaction with the PA protein of IAV or with different proteins, such as proteins with antiviral properties that can inhibit influenza virus replication and cell factors involved in IAV infection. The network of interactions during infection is very complex. We provide a new perspective for future investigations of these motifs, which will help obtain a more comprehensive insight into their role.

To date, neuraminidase inhibitors and M2 ion blockers are anti-influenza drugs that are globally approved and available for influenza infection (74). However, available drugs suffer from the rapid emergence of drug resistance because of influenza's high mutation rates (75); there is an urgent need to develop new antiviral strategies with new mechanisms of action and reduced drug resistance potential (74). Therefore, there is growing scientific interest in the host factors supporting viral infection. To achieve replication, RNA viruses are co-regulated with many cellular molecules: proteins, sugars, lncRNAs, lipids, hormones, and inorganic salts (74). Targeting the host factors was first used to control herpesvirus infection and is currently the major way of treating human immunodeficiency virus type 1 (HIV-1) infection (76). Nanoparticulate V-ATPase inhibitors were published against the influenza virus. It was shown that reduced viral titer in the mouse's lungs and short-term reduction of V-type ATPase activity in the lungs are tolerated by the host (77). Recently, a study targeting pre-mRNA and mRNA of TMPRSS2 protease that cleaves hemagglutinin (HA) using peptide-conjugated phosphorodiamide morpholino oligomers (PPMOs) and ASOs was published (78, 79). Thus, knowledge about the secondary structure of lncRNA-PAAN could be used to design an anti-influenza strategy. Our analysis of the lncRNA-PAAN secondary structure revealed a number of interesting structure targets. Using ASO tools, we inhibited IAV replication in cells, targeting the secondary structure motifs of lncRNA-PAAN. In the future, this strategy may be extended to create more effective inhibitors. To date, only a few studies (only bioinformatics prediction and bioinformatic prediction combined with mutagenesis study) addressed the structure of this host lncRNAs induced by IAV were performed (22, 23, 33, 66, 80). Research on the structure of lncRNAs induced during IAV infection and the inhibitory strategy based on structure is still in its early stages, and more work needs to be done.

## Experimental procedures

### Computational predictions of potential functional RNA structures of lncRNA-PAAN

For the computational predictions of lncRNA-PAAN structures, ScanFold 2.0 (45) was used (https://mosslabtools.bb.iastate.edu/scanfold2). It was exploited with a window size of 120 nucleotides, a step size of one nucleotide, and 100 mononucleotide randomization to compute the minimum free energy and the associated z-scores. The lncRNA-PAAN sequence was retrieved from the NCBI database (https://www.ncbi.nlm.nih.gov/gene/?term=LINC01988).

### DNA synthesis

The DNA template of lncRNA-PAAN was purchased from Integrated DNA Technologies Inc, (IDT). PCR reaction with primers FP, RP (Table S6) was used to produce the DNA template for *in vitro* transcriptions of lncRNA-PAAN. Primers 1FF and 1FR, 2FF and 2FR, 3FF and 3FR, 4FF and 4FR, and 5FF and 2FR (Table S6) were used to produce DNA templates for *in vitro* transcriptions of F1, F2, F3, F4, and F5 fragments. DNA was purified using the PCR/DNA Clean-Up Purification Kit from Eurx.

### Oligonucleotides synthesis and labeling

Primers for reverse transcription (Table S7) with fluorophores: 6-carboxyfluorescein (6-FAM) and 5-carboxy-4′,5′-dichloro-2′,7′-dimethoxyfluorescein (5-JOE) on the 5′-end and ASOs (Table S4) were synthesized by the phosphoramidite approach on a MerMade synthesizer. Oligonucleotides were deprotected and purified according to published protocols (81, 82). After purification, concentrations of all primers were measured using a UV Spectrophotometer (NanoDrop2000, Thermo Fisher Scientific). Primers for PCR and RT-qPCR (Tables S6 and S7) were purchased from Genomed.

### RNA synthesis

RNAs were obtained by transcription *in vitro* using a MEGAscript T7 Transcription Kit (Thermo Fisher Scientific) according to the manufacturer's protocol. RNA product was purified using the RNeasy MiniElute Cleanup Kit (Qiagen). The integrity and purity of samples were checked on an agarose gel.

### RNA folding

Before the *in vitro* mapping experiments, RNA was folded by heating it to 80 °C in water for 5 min and then slowly cooling it to 50 °C. Subsequently, a folding buffer (final concentration 300 mM NaCl, 5 mM MgCl_2_, 50 mM HEPES, pH 7.5) was added, and samples were slowly cooled to 37 °C. RNA integrity and homogeneity after folding were analyzed by native gel electrophoresis using a 0.8% agarose gel, run at 4 °C with a low voltage. Under these conditions, one band was observed.

### Cell culture and virus propagation

A549 cells (Merck, ECACC 86012804) were cultured in Dulbecco's modified Eagle's medium (DMEM; Thermo Fisher Scientific) supplemented with 10% heat-inactivated fetal bovine serum (FBS, Thermo Fisher Scientific), penicillin (100 U/ml), and streptomycin (100 μg/ml) (penicillin-streptomycin; Thermo Fisher Scientific) in 5% CO_2_ at 37 °C. A/California/04/2009 (H1N1) virus was a gift from Prof. Luis Martinez-Sobrido, Texas Biomedical Research Institute. Influenza A/California/04/2009 (H1N1) titers were determined using standard plaque assays (83, 84).

### Chemical mapping using NMIA, DMS, and CMCT

For *in vitro* experiments, RNA folding, as described above, was the first step in the mapping protocol. For mapping lncRNA-PAAN with ASO, the ASO was added before the folding step. Before the lysate mapping experiments, RNA (10 pmol) was heated for 5 min at 80 °C, cooled to 37 °C, and immediately added to the lysate for 30 min of incubation at 37 °C (85). Next, chemical mapping was performed according to published procedures with appropriate optimizations (86, 87, 88). Briefly, 5.6 mM of NMIA, 30 mM of CMCT, or 0.18% of DMS were used in *in vitro* mapping reactions. The lysate mapping experiments used a higher NMIA, CMCT, or DMS concentration (22 mM, 120 mM, and 0.75%). For DMS, CMCT, and NMIA probing, the reaction proceeded for 15 min, 30 min, and 40 min, respectively. Parallel control reactions were conducted in the same conditions but without mapping reagents. For experiments involving 18S rRNA and mapping lncRNA-PAAN in complex with ASOs, probing with NMIA and DMS was performed, as it allowed for comparative analysis. Mapping was carried out at 37 °C. The primer extinction reaction was exploited using a stoichiometry of 2 pmol primer/2 pmol RNA to read out modified nucleotides. For primer extinction reaction, reverse transcriptase Super Script III was exploited using the manufacturer's protocol. 6-FAM labeled primers were used to detect modification by DMS, CMCT, and NMIA, and in control reactions without mapping reagents. Reaction and control were resolved in the two capillaries with ddNTP ladders. For the ddNTP ladder, primers labeled with 5-JOE were used. The experiments were performed in at least technical triplicate, with the average results presented and the standard deviation (SD) calculated (Tables S1–S3 and S5).

### Processing of chemical mapping data

The QuShape program was used to analyze the mapping results. The mapping data was analyzed using a published method (89). The QuShape program normalized NMIA reactivities using model-free statistics to a scale spanning 0 to ∼2, where zero indicates no reactivity and 1.0 is the low average intensity for highly reactive RNA positions (89). Reactivities ≥ 0.7 were considered as strong, 0.7–0.5 were considered as medium, and ≤0.5 as weak. DMS and CMCT modifications analysis was performed similarly to NMIA reactivity calculations, except that only strong modifications (reactivities ≥ 0.7) were introduced in RNAstructure program prediction. Nucleotides with no data were designated as −999. For the prediction of lncRNA-PAAN, normalized SHAPE reactivities (as described above), as well as DMS and CMCT strong reactivities, were introduced in RNAstructure 6.4 through the “Read SHAPE reactivity—pseudo free energy” mode with a slope of 1.8 and an intercept of −0.6 kcal/mol and “chemical modification” mode, respectively (90, 91).

### Bioinformatic analysis of base pair probabilities

The lncRNA-PAAN base pair probabilities were obtained using the “Partition Function RNA” mode implemented in the RNA structure 6.4 program (56, 92). SHAPE, DMS, and CMCT reactivities were incorporated as constraints after loading the sequence file in “Partition Function RNA” mode, and a .pfs file was generated.

### Bioinformatic analysis of secondary structure ensembles

The Rsample program, available within the RNAstructure package, was used to stochastically sample lncRNA-PAAN secondary structures from the Boltzmann ensemble according to the publication (62). The IPANEMAP (Integrative Probabilistic Approaches for Nucleic Acid Ensemble Modeling and Analysis of Probing data) algorithm was used to produce probabilistic lncRNA-PAAN structure ensembles, as described in the publication (63).

### Preparation of lysates

Before infection, A549 cells were seeded at 3 × $10^{6}$ and grown until 90% confluence was reached. Diluted A/California/07/2009 (H1N1) virus in infection medium (0.3% BSA, 100 U/ml penicillin, 100 μg/ml streptomycin in DMEM) was added at 0.1 MOI. Cells were incubated for 1 h at 37 °C in 5% CO_2_ humidified incubator. After 1 h, the diluted virus was removed, and post-infection medium (0.3% BSA, 100 U/ml penicillin, 100 μg/ml streptomycin, 2 mM glutamine, and 1 μg/ml tosyl-sulfonyl phenylalanyl chloromethyl ketone (TPCK))-treated trypsin, supplemented with DMEM) was added. Cells were incubated in a post-infection medium for 24 h. Cells were then washed twice with 1xPBS and incubated with 2 ml of lysis buffer prepared according to a published protocol (93). Once complete, lysis was determined under the microscope and was detected by Lamin B1 and GADPH proteins. Lysates were divided and stored frozen at −80 °C.

### Western Blot

Lysates from infected A549 cells were checked for the presence of viral proteins (M2, NP), cytoplasmic fraction protein (GAPDH), and nuclear fraction protein (Lamin B1) by Western Blot. Lysates were lysed by Laemmli Sample Buffer with β-mercaptoethanol (Bio-Rad), incubated at 95 °C for 10 min, and separated by SDS-PAGE electrophoresis on a 4 to 15% gradient gel (Bio-Rad). Gels were transferred to a nitrocellulose membrane (Bio-Rad) and incubated overnight at 4 °C with a primary antibody. Next, membranes were incubated for 1 h at room temperature with HRP-labeled secondary antibodies. IAV protein expression was detected using rabbit anti-IAV NP (dilution 1:10,000), Thermo Fisher Scientific) and mouse anti-IAV M2 (dilution 1:10,000, Thermo Fisher Scientific) with HRP-conjugated anti-rabbit antibodies (dilution 1:100,000), Thermo Fisher Scientific) and anti-mouse secondary antibodies (dilution 1:100,000), Thermo Fisher Scientific), respectively. GAPDH expression was detected with a mouse anti-GAPDH monoclonal antibody (dilution 1:1000, Thermo Fisher Scientific) and HRP-conjugated anti-mouse antibodies (dilution 1:100,000, Thermo Fisher Scientific) served as loading controls, while Lamin B1 expression was detected with a mouse anti-lamin B1 monoclonal antibody (dilution 1:1000, Thermo Fisher Scientific) and HRP-conjugated anti-mouse antibodies (dilution 1:100,000, Thermo Fisher Scientific). The membranes were visualized by AZURE 400 Visible Fluorescent Imager.

### Coomassie brilliant blue staining

Using manufacturing protocols, total proteins from cell lysates were stained with Coomassie Brilliant Blue R-250 staining solution (Bio-Rad) (Fig. S6*A*).

### Oligonucleotide transfection

Cells were seeded in 24-well plates at 2 × 10^5^ cells/ml and incubated at 37 °C for 24 h. Transfection of ASOs was carried out using Lipofectamine 3000 (Thermo Fisher Scientific) according to the manufacturer's protocol. The final concentration of ASOs during transfection was 750 nM.

### Influenza virus infection

After 6 h of transfection, the cell cultures underwent a PBS wash and were infected with influenza virus A/California/04/2009 (H1N1) with an MOI of 0.5. Briefly, the cells were incubated with a solution containing virus diluted with infection medium (DMEM supplemented with 0.3% BSA [Sigma] and 100 U/ml penicillin and 100 μg/ml streptomycin [penicillin-streptomycin; Sigma]) for 1 h at 37 °C in 5% CO_2_ humidified incubator. After 1 h incubation, the supernatant was removed, and the cells were maintained in a postinfection medium (DMEM supplemented with 0.3% BSA, 100 U/ml penicillin, 100 μg/ml streptomycin, 2 mM glutamine, 1 μg/ml N-tosyl-L-phenylalanine chloromethyl ketone [TPCK]-treated trypsin [Sigma]) at 37°C. Cells were collected after 24 h.

### Quantitative real-time PCR analysis

The cells' lncRNA-PAAN and viral RNA copy numbers were determined by RT-qPCR. For this, total RNA from the cell monolayer was extracted using TRIzol reagent (94). DNase I treatment was performed according to the publication (95). Gene-specific primers (Table S7) complementary to the vRNA of five segments of the influenza A virus and random hexamers (Thermo Fisher Scientific) (for lncRNA-PAAN and GADPH) were used. According to the manufacturer's protocol, reverse transcription was performed with SuperScript III reverse transcriptase (Thermo Fisher Scientific). One microliter of the obtained cDNA was subjected to real-time PCR with gene-specific primers (Table S6) and 5× HOT FIREPol Probe qPCR Mix Plus (Solis BioDyne). Simultaneously, 1 μl of the obtained cDNA was subjected to real-time PCR to detect the GAPDH reference gene. The reaction was performed using the BIO-RAD CFX96 Real-Time system (Bio-Rad). The relative quantification in gene expression was determined using the 2^−ΔΔCt^ method (96). For absolute quantification of vRNA in infected cell lysates, we performed a reference standard. Briefly, we conducted *in vitro* transcription of vRNA 5. Next, the standards were reverse transcribed using SuperScript III, and serial 10-fold dilutions were prepared. The RNA from lysates was isolated and reverse-transcribed with SuperScript III enzyme according to the manufacturer's protocol. The real-time PCR reaction was performed as described above.

### Viability assay

A549 cells were seeded in 96-well plates at 2 × 10^5^ cells/ml density and incubated for 24 h at 37 °C and 5% CO_2_. Next, the cells were washed with PBS and transfected with ASOs, along with positive and negative controls. The cell viability was measured after 24 h using the CellTiter-Glo Assay (Promega) according to the manufacturer's protocol. The luminescence (200–300 nm) was measured using the Hidex Sense Plate Reader (Hidex).

### Shannon entropy calculation

Shannon Entropies were calculated from partition function data for local and global structure predictions. The per nucleotide Shannon entropy was calculated using the following:

$$S_{i}=-\sum_{j=1}^{N}P_{i,j}\;\cdot \log_{10}\left(P_{i.j}\right)$$

Where Si is the Shannon entropy for nucleotide i, i and j are nucleotide indices, and Pi,j is the probability of the i-j base-pair. Only valid i-j pairs are included. The N is the sequence length. Median Shannon Entropies were calculated for the center nucleotide in sliding 50 nt windows.

### Statistical analysis

Data are presented as mean ± standard deviation (SD) from at least three independent experiments. The normality of the data distribution was assessed using the Shapiro–Wilk test in GraphPad Prism 9. The data were normally distributed (*p* > 0.05). Statistical analyses were performed using a two-tailed Student's *t* test in GraphPad Prism 9. *p* values ≤ 0.05 were considered significant. ∗ denotes *p* ≤ 0.05, ∗∗ indicates *p* ≤ 0.01, ∗∗∗ implies ∗∗∗*p* < 0.005.

## Data availability

All data are contained within the article.

## Supporting information

This article contains supporting information.

## Conflict of interest

The authors declare that they have no conflicts of interest with the contents of this article.
