# Supplementary material for: Structural architecture of the human long non-coding RNA-PAAN as a potential target for anti-influenza drug development
Source: J Biol Chem. 2025 Sep 23;301(12):110755. doi: 10.1016/j.jbc.2025.110755 (PMC12651648; doi:10.1016/j.jbc.2025.110755)
Supplement: Supporting Figures [file mmc1.docx]

**Structural architecture of the human long non-coding RNA-PAAN, as a potential target for anti-influenza drug development**

Izabela Ulanowska ^1^, Ryszard Kierzek ^1^, Elzbieta Kierzek ^1^, Marta Soszynska-Jozwiak ^1*^

1 Institute of Bioorganic Chemistry, Polish Academy of Sciences, Noskowskiego 12/14, 61-704 Poznan, Poland

^*^ Author to whom correspondence should be addressed.

Figure S1. LncRNA-PAAN after folding by heating at 80°C for 5 min. in water and slowly cooled to 50°C. At 50°C buffer was added and slowly cooled to 37°C. Final folding buffer: 300mM NaCl, 5mM MgCl_2_, 50mM HEPES, pH 7.5.

Figure S2. MEA global structure of lncRNA-PAAN according to the experimental probing data in vitro. Visualization via PseudoViewer.

Figure S3. MEA local structure of lncRNA-PAAN mapped according to the experimental mapping data in vitro. Visualization via PseudoViewer.

Figure S4. Arc plots represent the secondary structures of lncRNA-PAAN in vitro predicted by Rsample. Visualization via R-chie .

Figure S5. Secondary structure of lncRNA-PAAN according to the experimental probing data in vitro generated by IPANEMAP. Visualization via PseudoViewer.

Figure S6. The IAV proteins expression in the infected cell lysates. A - the total protein from A549 cell lysates stained with Coomassie Brilliant Blue. B - the IAV protein expression was detected by Western blot analysis using anti-NP and anti-M2 antibodies. The presence of GADPH and Lamin B1, detected with anti-GADPH and anti-Lamin B1 antibodies, confirmed cellular and nuclear lysis, respectively. Expression of GADPH detected with anti-GADPH antibody served as a loading control. Protein was analyzed on a separate membrane. Only the lanes corresponding to relevant samples are shown. Splice positions (white gaps) indicate where non-adjacent lanes were removed from the original
