# Supplementary material for: Structural architecture of the human long non-coding RNA-PAAN as a potential target for anti-influenza drug development
Source: J Biol Chem. 2025 Sep 23;301(12):110755. doi: 10.1016/j.jbc.2025.110755 (PMC12651648; doi:10.1016/j.jbc.2025.110755)
Supplement: Supporting Figures [file mmc2.docx]

Figure S7. Amount of viral RNA5 copies in the independent biological replicates. The copies of vRNA5 were analyzed by RT-qPCR. Bars represent the mean ± standard deviation (SD) of n = 3 technical replicates. Each dot represents an independent technical replicate.

Figure S8. MEA global structure of lncRNA-PAAN according to the experimental data from probing in cell lysate. Visualization via PseudoViewer.

Figure S9. MEA local structure of lncRNA-PAAN according to the experimental data from mapping in cell lysate. Visualization via PseudoViewer.

Figure S10. Arc plots represent the secondary structures of lncRNA-PAAN in lysate predicted by Rsample. Visualization via R-chie.

Figure S11. Secondary structure of lncRNA-PAAN according to the experimental probing data in cell lysate generated by IPANEMAP. Visualization via PseudoViewer.

Figure S12. Cell viability treated/untreated with ASOs in a concentration of 0.75μM. The CellTiter-Glo Luminescent Cell Viability Assay (Promega) was utilized to assess cell viability 24 hours after treatment. Bars represent the mean ± standard deviation (SD) of n = 3 biological replicates. Each dot represents an independent biological replicate. The unpaired two-tailed Student’s t test was performed for statistical comparison (*p < 0.05, **p < 0.01, ***p < 0.005, ns- not significant).
